# Powers of County Medical Societies

**Published:** 1857-08

**Authors:** 


### EDITORIAL DEPARTMENT.

---

*Powers of County Medical Societies.*—With feelings of unusual pain and regret, we publish in this number the decision of Judge Marvin, of the Supreme Court, in the matter of the application of John D. Hill, to be restored to membership in the Erie County Medical Society.

We say unusual pain and regret, not that Dr. Hill should have gained by force of law a position which professional sentiment withheld from him, for once back in the society Dr. Hill is no longer the important individual which the cry of persecution and his prominent position as the contestant of the powers of a time-honored society have hitherto made him. He will naturally find his own level, and what that may be it is not for us to predict. The mere act of restoration is trivial and unimportant, and we, for one, care little about it.

But there is a principle involved here upon which, as we verily believe, hangs the life of this and every other county organization in the State, and it is a source of pain and regret that the hand of the judiciary should have stricken away the last vestige of all that was desirable in the chartered rights of the medical profession of the Empire State.

We have no great respect for that wondrous pile of circumlocution which is called law. It is a science founded on only human greed, and avarice, and trickery; on the assumption that all men are villians, so that when a question of honor comes up before it, honesty and straightforward purpose go to the wall before some merciless theory, simply because the law does not suppose the existence of honesty and straightforward purpose. Thus it was in this case. Judge Marvin recognizes the Erie County Medical Society as a corporation, and as a corporation he is ready to honor it. In his eyes a corporation has only one purpose, and that is to make money. The society in its tariff was trying to make money out of the public authorities, and Dr

Hill had a common-law right to make money in his own way which the society could not infringe. Consequently the rights of the corporation yield to those of the individual.

Now we had thought—we presume it would never occur to a judicial mind—that county societies were designed, and organized, and maintained, for the purpose of making men more honest than the law allows; of enforcing a higher code of morality and ethics than the law demands from the simple citizen. But according to Judge Marvin we have only the power to grant favors—none to exact conditions.

Those who read his argument carefully will discover something which may astonish them. The franchise of the society is so valuable, the respectability conferred by membership is so great, that it is beyond price; not to be paid for in personal sacrifice to the general good, the yielding of personal rights to the will of the majority. In fact, unless we read Judge Marvin wrong, we have no right to expel for anything less than a crime known to the law.

A society thus powerless is worthless. The only thing gained by our chartered organization was the privilege of choosing our company. The law—as we vainly thought—permitted us to set up a standard of scholarship and gentlemanly character, and draw a line of demarcation between those who practice medicine as a holy science and art, sacred and elevating in its tendencies, and that vile herd of pretenders to healing who work for gain only.

But the bench has decided otherwise. It has said that if we dare to check or thwart the money-getting spirit which now reigns supreme; dare to assert that medical services are not to be put up to the highest bidder, like cattle in the market, we shall be taught that we are powerless. Mammon is God! "The law declares it and the court so rules!"

We have one power left—yes, two. First, if a member steals or commits a rape—oh! what a compliment to ourselves is the supposition!—and we can prove it with all legal technicalities, we may—not expel him ourselves—but ask a county judge to do it for us! And, secondly, we can refuse admission to any one whom we please—thanks to Gov. Marcy, who, though dead, yet speaketh on that point. But we have no power of weeding our ranks. The smooth-faced young man comes to us with the wax still soft on the seal of his diploma, fair-spoken as to his intentions, and we admit him. Once in, there he is! He may reject all the rules of the code of ethics, cast from him every obligation which should bind the man of honor, and yet there he is, a member, and only some solemn county judge can make him anything else. He derives all the benefits of association on terms of open and declared equality with the most honored members of the profession, and requites those benefits by dragging them in the mire of his disgrace, while they have no power to cast him off. He may choose to practise open quackery, sneer at the time-honored profession which has named him "Doctor," and brazenly advertise to the town as a mountebank, a peddler of panaceas or of the vile concoctions of Thomsonianism. But we have no power to interefere, Judge Marvin has decided that the individual has an indefeasible right to get a living, honestly if he can, but at all events to get it.

Be it remembered that we are not impeaching the judicial wisdom of him who rendered this decisiou. Lawyers tell us that it is good law, and we believe it. We are only looking at the absurd position in which it places us poor men of the so-called regular profession, not in this case alone, for that is of small moment, but in all future time. We are coming down like law-loving citizens, to the question of dollars and cents, and inquiring what in the name of all that is decent is a County Society good for?

It is a convenient time now to stop and foot up our legal position and franchises—we like that last word, it is such a consolidated $10,000,000 capital sort of expression, implying a permanent investment somewhere.

There is a solemn statute in the books which Judge Marvin tells us all about, which once gave us a right to say who should and who should not be physicians, making the irregulars incompetent to collect their bills, and punishing them for manslaughter when their patients died; other rights it gave us, but as the goodly doctrine that one man is as good as another, and frequently a little better, gained stronger hold in the public mind; as our legislature became filled with men to whom this doctrine was particularly consoling, inasmuch as nothing short of legal enactment could make them as good as other men; one by one, our franchises—that magnanimous word—were stripped from us, until now the charlatan is the legal equal of the man of learning and position. The doors of a frantic liberalism were opened (and we are told that even the law got a taste of the same benefit at last, until even it is falling into contempt) antiquity, a glorious past and a present resplendent with learning, philanthropy and heroism in the face of new and strange pestilences, all the achievements of modern medicine were cast down and condemned to degradation as too slow—ah, too holy and too pure!—for this age of rapid progress and latter-day illumination. All this has been done through the gathered wisdom of Senate and Assembly by legislative enactment, and now the judiciary comes in to strip away the last vestige of our power, the only lingering remains of class distinction earned by how much patient heroism, by how many lives like those of the forty dead physicians of Norfolk, only the Last Day may tell.

The game is up. This decision has made the plaintiff a living member of a dead body, in that it strikes a deadly blow at the existence not only of this society, but of all other organizations in the state. We do not advocate the abandonment of these societies; that is unnecessary, they will die of this decision without further action. They are only useless excrescences on the healthy body of our profession. It—the profession—is sound and strong, all the law and all the lawyers cannot check its growth and usefulness. But voluntary association must take the place of chartered rights, and, placing ourselves in an equal field, we must fight out the battle with selfishness, pretension, and quackery on the sole ground of our own deservings.

